# ***Health in Humanitarian Emergencies: Principles and Practice for Public Health and Healthcare Practitioners*.** 2018. Edited by David Townes, Mike Gerber, and Mark Anderson. 485 pp. Cambridge University Press. ISBN 978-1-107-06268-9 Hardback

**DOI:** 10.4269/ajtmh.18-0839

**Published:** 2018-12

**Authors:** Karl Blanchet

**Affiliations:** Health in Humanitarian Crises Centre; London School of Hygiene & Tropical Medicine; London, United Kingdom; E-mail: karl.blanchet@lshtm.ac.uk

This is the first edition of *Health in Humanitarian Emergencies: Principles and Practice for Public Health and Healthcare Practitioners*. The book is a practical handbook, and it covers numerous important issues related to humanitarian emergencies. The editors have managed to keep the book quite condensed, at 485 pages. The book is quite heavy in the hardback version, but needs, nonetheless, to be packed in every suitcase and bag before going to the field. The book is also available in an electronic version. It is quite pricey, but aims to cover all the issues that relief workers are supposed to know when deployed to the field.

The book is organized into three sections subdivided into 31 chapters: 1) Humanitarian emergencies (six chapters), 2) public health principles (14 chapters), and 3) illness and injury (11 chapters). The choice for these three sections is not easy to understand. The first section is very clear, and describes the nature of emergencies, analyzing the various actors involved; different possible interventions; and the epidemiology of emergencies as ethical issues, which is a novel and relevant feature, considering current debates. However, the second section is a mix of chapters considering the various methods used (e.g., surveys, monitoring, and evaluation), areas of health intervention (e.g., nutrition and reproductive health), and support services (e.g., logistics). All the topics of health intervention could have been moved to the third section. The innovation is that a chapter on protection has been included in this health handbook. This sends a positive message to health professionals on the necessity to work more closely with protection professionals. The section on illness and injury is organized by illness and highlights a few acute conditions (e.g., acute respiratory infections and acute malnutrition).

The three editors of the book are from the U.S. Centers for Disease Control and Prevention (CDC), and quite a few chapter authors are from the same institution. The book was written by an impressive list of authors, who all have excellent reputations in their fields. A total of 81 authors contributed to the book. The authors are affiliated with various institutions in addition to the CDC, including United Nations agencies (e.g., WHO, the United Nations International Children’s Emergency Fund, United Nations High Commissioner for Refugees, and the United States Agency for International Development), American and European Universities, and one humanitarian organization, Médecins Sans Frontières. Being a practice handbook, the reader may have expected a few more authors from implementing agencies. However, all the authors have extensive experience working in this context and bring legitimacy to the book. The book offers a great opportunity to learn from CDC relief operations and have access to some of their training tools and instruments. Do not expect the book to touch on controversial issues. This book is about answering questions—practical ones—rather than opening debates.

The reader needs to go to page 3 and the introductory chapter to understand that an emergency becomes humanitarian, according to the editors, when it involves international organizations in the response. This is an interesting CDC perspective not based on a global consensus. This definition can be challenged based on the debates that occurred in 2016 at the World Humanitarian Summit in Istanbul emphasizing the importance of local actors in the humanitarian response.

There is a chapter on camp management, the content of which is fascinating, combining textual explanations with visual maps of camps. The reader will learn how the structure of a camp influences the well-being of its inhabitants and will influence how humanitarian assistance is delivered. To reflect on contemporary issues, the book could have added another chapter on non-camp management. For refugees who live in urban areas in standard housing, for example, it would have been interesting to provide guidance on the challenges posed to humanitarian organizations and national health authorities in making health services accessible in this kind of physical and social environment. This new situation, which has become more and more frequent in humanitarian settings, and more specifically in protracted crises, has forced relief organizations and national authorities to rethink the best way of delivering health care and ensuring a continuum of care.

The editors and authors have managed to keep every chapter synthetic, avoiding jargon and academic language, a writing style that fully corresponds to a practice guide. Almost every chapter makes great use of black and white visuals such as simple graphs, figures, and tables. They are all easy to understand and add great value to the text. These visuals can certainly be translated into training tools, which will help humanitarian professionals during operations. Each chapter is organized in short paragraphs and subsections with very clear headlines, which helps the reader to quickly navigate through the book and find the information needed in a minimum amount of time. The reader will have access to the latest evidence in each chapter and will be provided with practical tools to analyze a situation.

The list of references is an additional great resource. The editors took great pains to ensure that most resources listed in the book are open access. The reader can easily get access to them without going through a database or a university library. Again, this demonstrates the real purpose of this book: equipping humanitarian professionals with the latest evidence and tools.

*Health in Humanitarian Emergencies: Principles and Practice for Public Health and Healthcare Practitioners* is a great achievement, and it will become a classic in the next few years. There is no doubt that every humanitarian health professional should have this book on their desk when planning, implementing, and evaluating humanitarian interventions.

